# From Sensory Signals to Modality-Independent Conceptual Representations: A Probabilistic Language of Thought Approach

**DOI:** 10.1371/journal.pcbi.1004610

**Published:** 2015-11-10

**Authors:** Goker Erdogan, Ilker Yildirim, Robert A. Jacobs

**Affiliations:** 1 Department of Brain & Cognitive Sciences, University of Rochester, Rochester, New York, United States of America; 2 Department of Brain & Cognitive Sciences, Massachusetts Institute of Technology, Cambridge, Massachusetts, United States of America; 3 Laboratory of Neural Systems, The Rockefeller University, New York, New York, United States of America; University of Minnesota, UNITED STATES

## Abstract

People learn modality-independent, conceptual representations from modality-specific sensory signals. Here, we hypothesize that any system that accomplishes this feat will include three components: a representational language for characterizing modality-independent representations, a set of sensory-specific forward models for mapping from modality-independent representations to sensory signals, and an inference algorithm for inverting forward models—that is, an algorithm for using sensory signals to infer modality-independent representations. To evaluate this hypothesis, we instantiate it in the form of a computational model that learns object shape representations from visual and/or haptic signals. The model uses a probabilistic grammar to characterize modality-independent representations of object shape, uses a computer graphics toolkit and a human hand simulator to map from object representations to visual and haptic features, respectively, and uses a Bayesian inference algorithm to infer modality-independent object representations from visual and/or haptic signals. Simulation results show that the model infers identical object representations when an object is viewed, grasped, or both. That is, the model’s percepts are modality invariant. We also report the results of an experiment in which different subjects rated the similarity of pairs of objects in different sensory conditions, and show that the model provides a very accurate account of subjects’ ratings. Conceptually, this research significantly contributes to our understanding of modality invariance, an important type of perceptual constancy, by demonstrating how modality-independent representations can be acquired and used. Methodologically, it provides an important contribution to cognitive modeling, particularly an emerging probabilistic language-of-thought approach, by showing how symbolic and statistical approaches can be combined in order to understand aspects of human perception.

## Author Summary

When viewing an object, people perceive the object’s shape. Similarly, when grasping the same object, they also perceive its shape. In general, the perceived shape is identical in these two scenarios, illustrating modality invariance, an important type of perceptual constancy. Modality invariance suggests that people infer a modality-independent, conceptual representation that is the same regardless of the modality used to sense the environment. If so, how do people infer modality-independent representations from modality-specific sensory signals? We present a hypothesis about the components that any system will include if it infers modality-independent representations from sensory signals. This hypothesis is instantiated in a computational model that infers object shape representations from visual or haptic (i.e., active touch) signals. The model shows perfect modality invariance—it infers the same shape representations regardless of the sensory modality used to sense objects. The model also provides a highly accurate account of data collected in an experiment in which people judged the similarity of pairs of objects that were viewed, grasped, or both. Conceptually, our research contributes to our understanding of modality invariance. Methodologically, it contributes to cognitive modeling by showing how symbolic and statistical approaches can be combined in order to understand aspects of human perception.

## Introduction

While eating breakfast, you might see your coffee mug, grasp your coffee mug, or both. When viewing your mug, your visual system extracts and represents the shape of your mug. Similarly, when grasping your mug, your haptic system also extracts and represents the shape of your mug. Are the representations acquired when viewing your mug distinct from the representations acquired when grasping your mug? If so, these would be modality-specific representations. Or does there exist a level at which the shape representation of your mug is the same regardless of the sensory modality through which the mug is perceived? If so, this would be a modality-independent representation.

Recent experiments on crossmodal transfer of perceptual knowledge suggest that people have multiple representations of object shape and can share information across these representations. For example, if a person is trained to visually categorize a set of objects, this person will often be able to categorize novel objects from the same categories when objects are grasped but not seen [1, 2]. Because knowledge acquired during visual training is used during haptic testing, this finding suggests that neither the learning mechanisms used during training nor the representations acquired during training are exclusively visual. To the contrary, the finding indicates the existence of both visual and haptic object representations as well as the ability to share or transfer knowledge across these representations. Successful categorization of objects regardless of whether the objects are seen or grasped illustrates modality invariance, an important type of perceptual constancy.

What type of learning mechanisms and mental representations might underlie modality invariance? One possible answer is that people are able to abstract over their modality-specific representations in order to acquire modality-independent representations. For instance, people might use modality-specific representations of objects as a foundation for inferring modality-independent representations characterizing objects’ intrinsic properties. To understand the nature of the latter representations, it is important to recognize the distinction between objects’ intrinsic (or “deep”) properties and the sensory (or “surface”) features that these properties give rise to. The shape of an object is a modality-independent intrinsic property. Visual and haptic features are modality-specific sensory cues to the object’s shape arising when the object is viewed or grasped, respectively.

Once acquired, modality-independent representations may underlie modality invariance. For example, they can mediate crossmodal transfer of knowledge. Consider a person who is first trained to visually categorize a set of objects, and then tested with novel objects (from the same set of categories) when the objects are grasped but not seen. During visual training, the person uses his or her visual representation of each object to infer a modality-independent representation characterizing the object’s intrinsic properties, and applies the object’s category label to this representation. When subsequently grasping a novel object on a test trial, the person uses the object’s haptic representation to infer a modality-independent representation of its intrinsic properties. The novel object is judged to be a member of a category if it has similar intrinsic properties to the training objects belonging to that category.

Because modality-independent representations may underlie modality invariance, they would clearly be useful for the purposes of perception and cognition. Importantly, recent behavioral and neurophysiological data indicate their existence in biological organisms. For instance, behavioral and neural evidence support the idea that object features extracted by vision and by touch are integrated into modality-independent object representations that are accessible to memory and higher-level cognition [3–15]. Based on brain imaging (fMRI) data, Taylor et al.[10] argued that posterior superior temporal sulcus (pSTS) extracts pre-semantic, crossmodal perceptual features, whereas perirhinal cortex integrates these features into amodal conceptual representations. Tal and Amedi [15], based on the results of an fMRI adaptation study, claimed that a neural network (including occipital, parietal, and prefrontal regions) showed crossmodal repetition-supression effects, indicating that these regions are involved in visual-haptic representation.

Perhaps the most striking data comes from the work of Quiroga and colleagues who analyzed intracranial recordings from human patients suffering from epilepsy [16, 17]. Based on these analyses, they hypothesized that the medial temporal lobe contains “concept cells”, meaning neurons that are selective for particular persons or objects regardless of how these persons or objects are sensed. For instance, Quiroga et al [16] found a neuron that responded selectively when a person viewed images of the television host Oprah Winfrey, viewed her written name, or heard her spoken name. (To a lesser degree, the neuron also responded to the comedian Whoopi Goldberg.) Another neuron responded selectively when a person saw images of the former Iraqi leader Saddam Hussein, saw his name, or heard his name.

To fully understand modality-independent representations, Cognitive Science and Neuroscience need to develop theories of how these representations are acquired. Such theories would be significant because they would help us understand the relationships between perceptual learning and modality invariance. They would also be significant because they would be early “stepping stones” toward developing an understanding of the larger issue of how sensory knowledge can be abstracted to form conceptual knowledge.

The plan of this paper is as follows. In the Results section, we start by describing a general theoretical framework for how modality-independent representations can be inferred from modality-specific sensory signals. To evaluate the framework, we next describe an instantiation of the framework in the form of a computational model, referred to as the Multisensory-Visual-Haptic (MVH) model, whose goal is to acquire object shape representations from visual and/or haptic signals. Simulation results show that the model learns identical object representations when an object is viewed, grasped, or both. That is, the model’s object percepts are modality invariant. We also evaluate the MVH model by comparing its predictions with human experimental data. We report the results of an experiment in which subjects rated the similarity of pairs of objects, and show that the model provides a very successful account of subjects’ ratings. In the Discussion section, we highlight the contributions of our theoretical framework in general, and of the MVH model in particular, emphasizing its combination of symbolic and statistical approaches to cognitive modeling. Due to this combination, the model is consistent with an emerging “probabilistic language of thought” methodology. The Methods section provides modeling and experimental details.

## Results

### Theoretical framework

According to our framework, any system (biological or artificial) that acquires modality-independent representations from sensory signals will include the following three components: (1) a representational language for characterizing modality-independent representations; (2) sensory-specific forward models for mapping from modality-independent representations to sensory signals; and (3) an inference algorithm for inverting sensory-specific forward models—that is, an algorithm for using sensory signals in order to infer modality-independent representations. These three components are discussed in turn.

#### (1) Representational language for characterizing modality-independent representations

Although biological representations of modality-specific sensory signals are not fully understood, it is believed that these representations are constrained by the properties of the perceptual environment and the properties of the sensory apparatus. For example, the nature of biological visual representations depends on the nature of the visual environment and the nature of the eye.

In contrast, constraints on the nature of modality-independent representations are not so easy to identify. One radical view, usually referred to as embodied cognition [18], claims that there are no amodal representations; all mental representations consist of sensory representations. However, the majority view in Cognitive Science argues that people possess modality-independent representations (e.g., representations of object shape or representations of abstract concepts such as ‘love’ or ‘justice’), though there is no consensus as to the best way to characterize these representations. Common approaches include both statistical (e.g., distributed representations over latent variables) and symbolic (e.g., grammars, logic) formalisms. These formalisms provide different representational languages for expressing modality-independent thoughts and ideas, each with their own strengths and weaknesses.

#### (2) Sensory-specific forward models

Modality-independent representations do not make direct contact with sensory signals. To bring them in contact with sensory signals, our framework includes sensory-specific forward models which map from modality-independent representations to sensory features. For example, a vision-specific forward model might map a modality-independent representation of an object’s shape to an image of the object when viewed from a particular viewpoint. Similarly, a haptic-specific forward model might map the same modality-independent representation of an object’s shape to a set of haptic features (e.g., hand shape as characterized by the joint angles of a hand) that would be obtained when the object is grasped at a particular orientation. We often find it useful to think of these sensory-specific forward models as implementations of sensory imagery. For instance, if a vision-specific forward model maps an object to its visual features, then that is an implementation of visual imagery.

#### (3) Inference algorithm for inverting forward models

Sensory-specific forward models map from modality-independent representations to sensory signals. However, perception operates in the opposite direction—it maps from sensory signals to modality-independent representations. Consequently, perception needs to invert the sensory-specific forward models. This inversion is accomplished by a perceptual inference algorithm.

From a larger perspective, our theoretical framework presents a conceptual analysis of the computational problem of multisensory perception. How can we transfer knowledge (category, shape, meaning etc.) from one modality to another? Why are we more accurate when we perceive through more modalities? How can we recognize a novel object crossmodally? Or how can we recognize an object crossmodally from a novel view? We believe our framework is successful in providing a unified account of the answers to these questions and the underlying cognitive processes. Hence, we believe our theoretical framework in itself constitutes a significant contribution to the understanding of multisensory perception.

### Framework applied to visual-haptic object shape perception

To better understand and evaluate our framework, we apply it to the perception of object shape via visual and haptic modalities. This application results in the MVH computational model with the three components outlined above.

We have had to make specific implementation choices to instantiate our theoretical framework as a computational model. To us, these choices are both uninteresting and interesting. On the one hand, the implementation choices that we have made are not essential to the framework. Indeed, other reasonable choices could have been made, thereby leading to alternative framework implementations. On the other hand, we believe that some of our choices are important because they contribute to the study of cognitive modeling. In particular, our computational model combines both symbolic and statistical modeling approaches. Because of this combination, the model can be regarded as falling within a recently emerging “probabilistic language of thought” methodology. This contribution is described in the Discussion section.

One of the implementation choices that we made was a choice as to which stimuli we should focus on. Object shape perception via vision and/or haptics is currently an unsolved problem when considered in its full generality. Consequently, we focus on a small subset of objects. We designed 16 novel objects, where the set of object parts was based on a previously existing set of objects known as “Fribbles”. Fribbles are complex, 3-D objects with multiple parts and spatial relations among parts. They have been used in studies of visual [19, 20] and visual-haptic [2] object perception. We used part-based objects because many real-world objects (albeit not all) have a part-based structure. In addition, theories of how people visually recognize part-based objects have received much attention and played important roles in the field of Cognitive Science [21–25].

**Fig 1:**
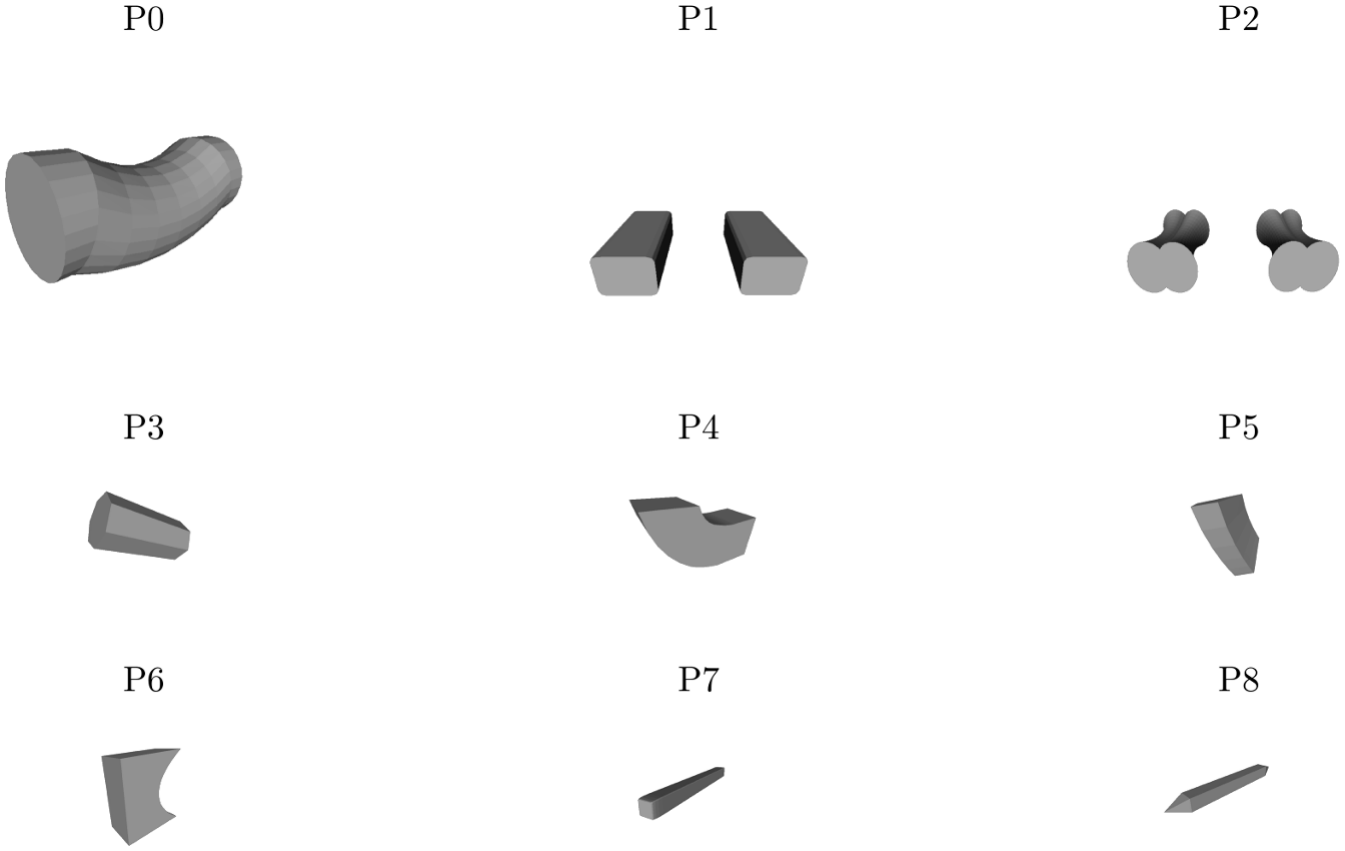
Possible object parts. Part P0 is common to all objects. Parts P1–P8 vary from object to object.

**Fig 2:**
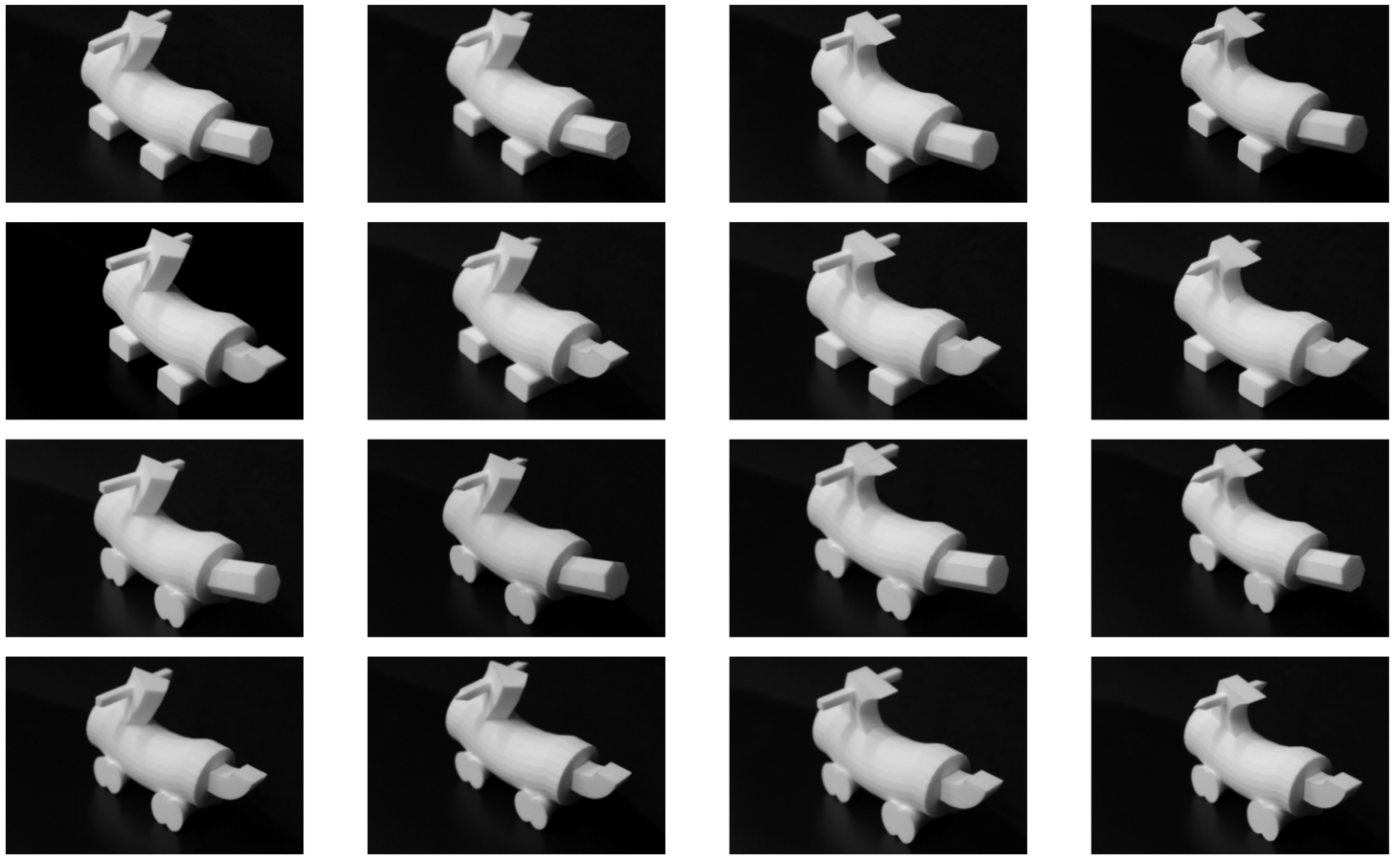
Images of objects used in our simulations and experiment.

Each object that we designed is comprised of five parts (the set of possible parts is shown in Fig 1). One part (labeled P0 in Fig 1), a cylindrical body, is common to all objects. The remaining four parts vary from object to object, though they are always located at the same four locations in an object. A particular object is specified by selecting one of two interchangeable parts at each location (4 locations with 2 possible parts per location yields 16 objects). The complete set of objects is shown in Fig 2.

#### Shape grammar as a language for characterizing object shape

In the MVH model, object representations have three important properties. The first property is that representations are modality-independent. That is, they are not directly composed from modality-specific features, nor do they directly specify the values of these features.

The second property is that object representations characterize objects in terms of their parts and the spatial relations among these parts. When designing the model, our main focus was not on developing new insights regarding how people represent object shape. Although this is an important research area, many researchers in the Cognitive Science and Artificial Intelligence communities already study this topic [21–35]. The scientific literature contains a wide variety of different approaches to object shape representation. To date, there does not appear to be a consensus as to which approach is best.

Instead of researching new ways to represent object shape, our goal is to understand how modality-independent representations can be learned from sensory data. Because the MVH model needs to represent object shape, it necessarily resembles previously existing models that also represent object shape. In particular, like previous models, our model represents objects in terms of their parts and the spatial relations among these parts [21–25]. In principle, we are not strongly committed to the hypothesis that people represent objects in a part-based manner. Shape primitives other than parts could have been used in our simulations (as is sometimes done with shape grammars in the Computer Vision and Computer Graphics literatures; e.g., see [36]), albeit at possibly greater computational expense. To us, the use of part-based object representations in our simulations seems reasonable because these representations have played prominent roles and received considerable theoretical and empirical support in the Cognitive Science literature, because the stimuli used in our simulations and experiment were generated in a part-based manner, because the analyses of our experimental data indicate that subjects were sensitive to the part-based structure of the stimuli (see below), and because part-based object representations led to computationally tractable simulations.

The final property is that object representations use a shape grammar to characterize an object’s parts and the spatial relations among these parts [35–42]. Grammars are commonly used to characterize human language and language processing [43, 44], and are also used in other areas of Cognitive Science [45–50]. In addition, they are used to characterize objects and scenes in fields such as Computer Vision and Computer Graphics [35–42].

The MVH model uses a shape grammar to specify the possible parts and spatial relations among parts. Conventional shape grammars, like other types of symbolic representations, can often be “brittle” when used in noisy environments with significant uncertainty. We ameliorated this problem through the use of a probabilistic approach. The details of the shape grammar are described in the Methods section. For now, note that the grammar is an instance of a probabilistic context-free grammar. Production rules characterize the number of parts and the specific parts comprising an object. These rules are supplemented with information characterizing the spatial relations among parts.

Specifically, an object is generated using a particular sequence of production rules from the grammar. This sequence is known as a derivation which can be illustrated using a parse tree. To fully specify an object, an object’s derivation or parse tree is supplemented with information specifying the locations of the object’s parts. This specification occurs by adding extra information to a parse tree, converting this tree to a spatial tree representing object parts and their locations in 3-D space (see Methods section).

#### Vision-specific and haptic-specific forward models

Because object representations are modality independent, they do not make direct contact with sensory signals. To evaluate and infer these representations, they need to be brought in contact with these signals. For these purposes, the MVH model uses its modality-independent representations to predict or “imagine” sensory features from individual modalities. For example, given a modality-independent representation of a particular object (i.e., a representation of the object’s parts and the locations of these parts), the model can predict what the object would look like (perhaps a form of visual imagery) or predict the hand shape that would occur if the object were grasped (perhaps a form of haptic imagery). A mapping from a modality-independent representation to a sensory-specific representation can be carried out by a forward model, a type of predictive model that is often used in the study of perception and action [51–53]. In Cognitive Science, forward models are often mental or internal models. However, forward models exist in the external world too. Our computer simulations made use of two forward models.

The vision-specific forward model was the Visualization Toolkit (VTK; www.vtk.org), an open-source, freely available software system for 3-D computer graphics, image processing, and visualization. We used VTK to visually render objects. Given a modality-independent representation of an object, VTK rendered the object from three orthogonal viewpoints. Images were grayscale, with a size of 200 × 200 pixels. A visual input to the model was a vector with 120,000 elements (3 images × 40,000 [200 × 200] pixels per image).

**Fig 3:**
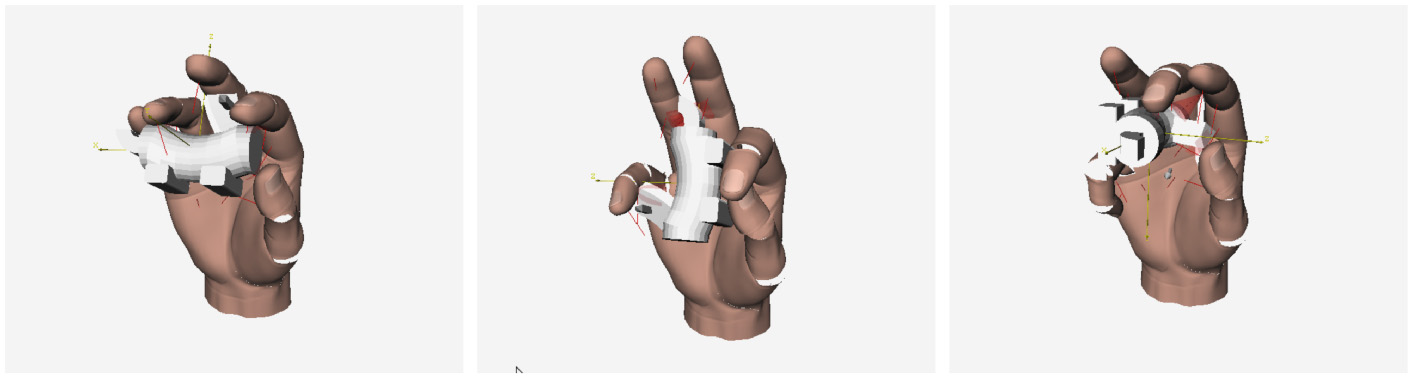
GraspIt! simulates a human hand. Here the hand is grasping an object at three different orientations.

The haptic-specific forward model was a grasp simulator known as “GraspIt!”[54]. GraspIt! contains a simulator of a human hand. When predicting the haptic features of an object, the input to GraspIt! was the modality-independent representation for the object. Its output was a set of 16 joint angles of the fingers of a simulated human hand obtained when the simulated hand “grasped” the object. Grasps—or closings of the fingers around an object—were performed using GraspIt!’s AutoGrasp function. Fig 3 shows the simulated hand grasping an object at three orientations. In our simulations, each object was grasped 24 times, each time from a different orientation (different orientations were generated by rotating an object 8 times [each time by 45°] around the width, length, and depth axes). The use of multiple grasps can be regarded as an approximation to active haptic exploration. A haptic input to the model was a vector with 384 elements (16 joint angles per grasp × 24 grasps). Our choice of using joint angles as our haptic features follows a common practice in the field of postural hand analysis [55, 56].

#### Bayes’ rule inverts sensory-specific forward models

Importantly, the MVH model learns its object representations. The most influential models of object shape in the Cognitive Science literature, such as those of Biederman [23] and Marr and Nishihara [21], used part-based shape representations that were stipulated or hand-crafted by scientific investigators. In contrast, a goal of our model is to learn representations using a probabilistic or Bayesian inference algorithm from visual and/or haptic signals. Using the terminology of Bayesian inference, the model computes a posterior distribution over object representations based on a prior distribution over these representations (indicating which of the representations are more or less likely before observing any sensory data) and a likelihood function (indicating which representations are more or less likely to give rise to observed sensory data).

The model’s prior distribution is based on the prior distribution of the Rational Rules model of Goodman et al.[45]. In brief (see the Methods section for full details), the prior distribution is the product of two other distributions, one providing a prior over parse trees and the other providing a prior over spatial models. These priors are Occam’s Razors favoring the use of “simple” parse trees and spatial models.

The likelihood function allows the model to use sensory data to evaluate proposed object representations. Object representations which are highly likely to give rise to perceived sensory data are more probable than object representations which are less likely to give rise to these data (ignoring the prior distribution, for the moment). Sensory-specific forward models play a crucial role in this evaluation. As mentioned above, object representations are modality-independent, and thus do not make direct contact with perceived visual or haptic features. Sensory-specific forward models are needed to relate object representations to their sensory features.

Using Bayes’ rule, the MVH model combines the prior distribution and the likelihood function to compute a posterior distribution over object representations. Unfortunately, exact computation of the posterior distribution is intractable. We, therefore, developed a Markov chain Monte Carlo (MCMC) algorithm that discovers good approximations to the posterior. This algorithm is described in the Methods section.

#### Simulation results

We used the model to infer modality-independent representations of the 16 objects in Fig 2. Object representations were inferred under three stimulus conditions: a vision condition, a haptic condition, and a multisensory (visual and haptic) condition. In all conditions, we inferred the posterior distribution over modality-independent object representations. However, except where explicitly noted, the results reported below are based on maximum a posteriori (MAP) estimates. Because distributions are highly peaked around the MAP estimate, the results are essentially the same when samples from each distribution are used.

The sole free parameter of the model is the variance of the likelihood function. Intuitively, this parameter controls the relative weights of the prior and likelihood terms. By increasing the variance, thereby increasing the relative weight of the prior, it is possible to constrain the model so that it tends to prefer simple parse trees and spatial models. In contrast, as the variance is decreased, the likelihood becomes more important, thus allowing more complex trees and models to be assigned probability mass. For each stimulus condition, we selected a value for the variance that provides a good balance between prior and likelihood terms. We found that simulation results are robust to the exact choice for the variance value. As long as the variance is small enough to allow object representations which are complex enough, the MVH model produced similar results.

**Fig 4:**
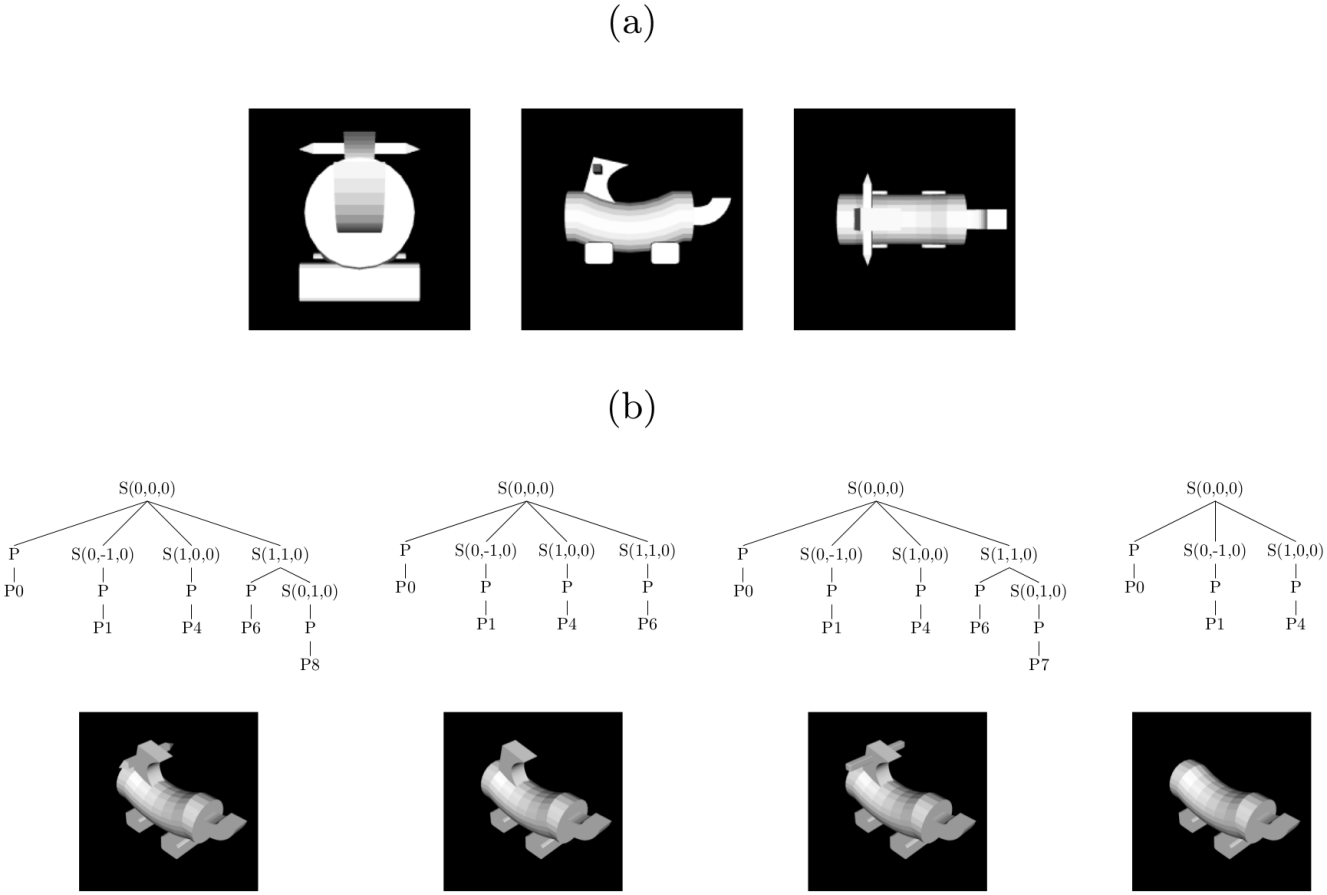
Results from a representative simulation of the MVH model. (a) Visual input to the model. (b) Four estimates of modality-independent object representations (parse trees augmented with spatial information) with the highest posterior probabilities (top) and their images (bottom). The MAP estimate is on the left. Each S (spatial) node denotes a position in 3D space relative to its parent S node. P (part) nodes specify the part located at its parent S node position. For example, in all the trees here P0 is located at its ancestor S node’s position, which is the origin. The depth of a P node corresponds roughly to its distance from the origin. Please refer to Methods for more details.

Fig 4 shows the results of a representative simulation in which the model received visual input. This input consisted of three images of an object from orthogonal viewpoints (Fig 4a). The four modality-independent object representations with the highest posterior probabilities are shown in the top row of Fig 4b. The bottom row shows visual renderings of these object representations. The MAP estimate is on the left. Crucially, this estimate represents the object perfectly, successfully inferring both the object parts and their spatial locations. Indeed, we find that the model’s MAP estimate always represents an object perfectly for all the objects comprising our stimulus set.

The other estimates in Fig 4b (estimates with smaller probabilities than the MAP estimate) exemplify the robustness of the model. Although imperfect, these estimates are still sensible. When a part is missing from an object representation, it is often part P8 which is small in size and, thus, has only a small influence on the likelihood function. When a part is mismatched, the model often substitutes part P7 for P8. This is unsurprising given that parts P7 and P8 are visually (and haptically) similar.

Critically, the model shows perfect modality invariance. That is, it performs identically in vision, haptic, and multisensory conditions, meaning the model produces the same MAP estimate of an object’s parts and spatial relations among these parts regardless of whether the object is viewed, grasped, or both. For example, if the model is given the haptic features of the object shown in Fig 4a (instead of images of the object), its MAP estimate is still the parse tree on the left of Fig 4b. This result demonstrates that the object representations acquired by the model are modality independent. For this reason, we do not discuss separately the model’s performances in vision, haptic, and multisensory conditions—these performances are identical.

### Comparison with human data

Above, the motivations and merits of our computational model were described based primarily on theoretical grounds. Here, we evaluate the MVH model based on its ability to provide an account of human experimental data. The experiment reported here is related to the experiments of Wallraven, Bülthoff, and colleagues who asked subjects to rate the similarity of pairs of objects when objects were viewed, grasped, or both [57–61]. However, our experiment also includes a crossmodal condition in which subjects rated object similarity when one object was viewed and the other object was grasped.

In brief (experimental details are given in the Methods section), the stimuli were the 16 objects described above (Fig 2). On each trial, a subject observed two objects and judged their similarity on a scale of 1 (low similarity) to 7 (high similarity). The experiment included four conditions referred to as the visual, haptic, crossmodal, and multisensory conditions. Different groups of subjects were assigned to different conditions. In the visual condition, subjects viewed images of two objects on each trial. In the haptic condition, subjects grasped physical copies of two objects (fabricated using 3-D printing) on each trial. In the crossmodal condition, subjects viewed an image of one object and grasped a second object on each trial. Finally, in the multisensory condition, subjects viewed and grasped two objects on each trial.

#### Experimental results

If people’s perceptions of object shape are modality invariant, then subjects in all conditions should perform the experimental task in the same manner: On each trial, a subject represents the intrinsic shape properties of the two observed objects in a modality-independent format, and then the two modality-independent object representations are compared to generate a similarity judgment. The goal of the analyses of our experimental data is to evaluate whether subjects in fact based their similarity judgments on modality-independent shape representations. We look at this question by testing various predictions of the modality-invariance hypothesis. First, if people’s perceptions of object shape are modality invariant, their similarity judgments should be quite similar regardless of modality. Hence, one would expect to see high correlations between similarity judgments not only within conditions but also across conditions. We test this prediction with our first analysis below. A much stronger test of modality invariance is possible if we can somehow find the shape representations subjects employed in each condition. We can then simply compare these representations to evaluate modality invariance. In our second set of analyses, we use additive clustering and Multidimensional Scaling (MDS) to infer the perceptual space for each condition and compare them.

First, we looked at the average of subjects’ similarity ratings for identical objects; this provides us with a coarse measure of modality invariance as well as a measure of objective accuracy. As expected from modality invariant representations, these ratings were nearly 7 (Visual: 6.89±0.27, Haptic: 6.74±0.47, Crossmodal: 6.71±0.49, Multisensory: 6.82±0.35). To address the question of modality invariance further, we proceeded as follows. First, for each subject in our experiment, we formed a subject-level similarity matrix by averaging the subject’s ratings for each pair of objects. Next, we correlated a subject’s similarity matrix with the matrices for subjects in the same experimental condition and in other conditions. The average correlations are shown in Table 1. These correlations are large, ranging from 0.76 to 0.91 (explaining 58%–83% of the variance in subjects’ ratings). To test if these values are significantly greater than zero, we transformed them using the Fisher *z*-transformation. A *t*-test using the transformed correlations indicated that all correlations are significantly greater than zero (p < 0.001 in all cases). We are primarily concerned with whether subjects from different conditions gave similar similarity ratings, and thus we closely examined the average correlations when subjects were in different conditions (for example, cells Visual-Haptic or Visual-Crossmodal, but not Visual-Visual or Haptic-Haptic, in the matrix in Table 1). Using *t*-tests, we asked if each of these correlations is “large”, which we (arbitrarily, but not unreasonably) defined as meaning that a correlation explains at least 50% of the variance in subjects’ ratings. All of these correlations were found to be large by this definition (p < 0.001 in all cases). Lastly, for each condition, we also formed a condition-level similarity matrix by averaging the subject-level matrices for the subjects belonging to that condition. As shown in Table 2, correlations among these condition-level matrices were extremely high, with the smallest correlation equal to 0.97 (explaining 94% of the variance in subjects’ ratings across conditions). Taken as a whole, our correlational analyses strongly suggest that subjects had similar notions of object similarity in all experimental conditions. In other words, subjects’ similarity ratings were modality invariant.

**Table 1:** Average correlations within and across conditions among subjects’ similarity matrices.

	Visual	Haptic	Crossmodal	Multisensory
Visual	0.91 ± 0.0341	0.83 ± 0.0855	0.86 ± 0.0729	0.89 ± 0.0572
Haptic		0.76 ± 0.1032	0.78 ± 0.1052	0.81 ± 0.0962
Crossmodal			0.8 ± 0.0968	0.83 ± 0.0866
Multisensory				0.86 ± 0.0651

For example, the value in the Visual-Visual cell was calculated by averaging over correlations in subjects’ similarity ratings for each pair of subjects in the visual condition (because there were 7 subjects in this condition, there were 42 = 7 × 6 such pairs). Similarly, the value in the Visual-Haptic cell was calculated by averaging over correlations for each pair of subjects when one subject was in the visual condition and the other subject was in the haptic condition (because there were 7 subjects in each condition, there were 49 such pairs).

**Table 2:** Correlations based on condition-level similarity matrices formed by averaging subject-level matrices for the subjects belonging to each condition.

	Visual	Haptic	Crossmodal	Multisensory
Visual	0.99 ± 0.0072	0.95 ± 0.0206	0.96 ± 0.0138	0.97 ± 0.0134
Haptic		0.97 ± 0.0241	0.94 ± 0.023	0.94 ± 0.0232
Crossmodal			0.97 ± 0.0199	0.96 ± 0.017
Multisensory				0.98 ± 0.0136

Means and standard deviations are estimated with a bootstrap procedure with 1000 replications.

We further analyzed the experimental data using a Bayesian nonparametric additive clustering technique due to Navarro and Griffiths [62]. This technique makes use of the Indian Buffet Process [63], a latent feature model recently introduced in the Machine Learning and Statistics literatures. In brief, the technique infers the latent or hidden features of a set of stimuli from their similarities. In our context, the technique assumes that subjects’ similarity ratings are generated from hidden or latent binary object representations. Using Bayes’ rule, it inverts this generative process so that similarity ratings are used to infer probability distributions over object representations. In other words, the input to the technique is a matrix of similarity ratings. Its output is a probability distribution over object representations where representations that are likely to give rise to the similarity ratings are assigned higher probabilities. The dimensionality of the binary object representations is not fixed. Rather, the technique infers a probability distribution over this dimensionality.

We applied the technique to each of the condition-level similarity matrices. In all conditions, it revealed that the most probable dimensionality was eight (i.e., similarity ratings in all conditions were most likely based on object representations consisting of eight binary features). However, the technique inferred two identical copies of each dimension, a potential problem noted by Navarro and Griffiths [62]. Consequently, the technique actually inferred four-dimensional object representations in all conditions. Interestingly, these object representations can be interpreted as “part based” representations of our experimental stimuli. Recall the structure of the experimental objects. There are four locations on objects at which parts vary. At each location, there are two interchangeable parts, only one of which is present in a given object. As a matter of notation, label the first set of interchangeable parts as {P1, P2}, the second set as {P3, P4}, and so on. An object can, therefore, be represented by four binary numbers. One number indicates which part is present in the set {P1, P2}, another number indicates which part is present in the set {P3, P4}, etcetera. We refer to this as a list-of-parts object representation.

The Bayesian nonparametric additive clustering technique inferred the same list-of-parts object representation as its MAP estimate when applied to every condition-level similarity matrix. This is important because it suggests that the same object representations underlied subjects’ similarity ratings in visual, haptic, crossmodal, and multisensory experimental conditions. That is, this analysis of our data suggests that subjects used modality-independent representations, and thus our data are consistent with the hypothesis that subjects’ object perceptions were modality invariant. Importantly, the result did not have to come out this way. If the additive clustering technique inferred different object representations when applied to different condition-level similarity matrices, this outcome would have been inconsistent with the hypothesis of modality invariance.

The fact that the additive clustering technique always inferred *part-based* representations is also noteworthy. In hindsight, however, it might be unsurprising for subjects to have used part-based representations. Recall that our stimuli were generated by combining distinct parts. It seems likely that subjects would be sensitive to the structure of this generative process. Moreover, previous theoretical and empirical studies have indicated that people often use part-based object representations [21–25].

**Fig 5:**
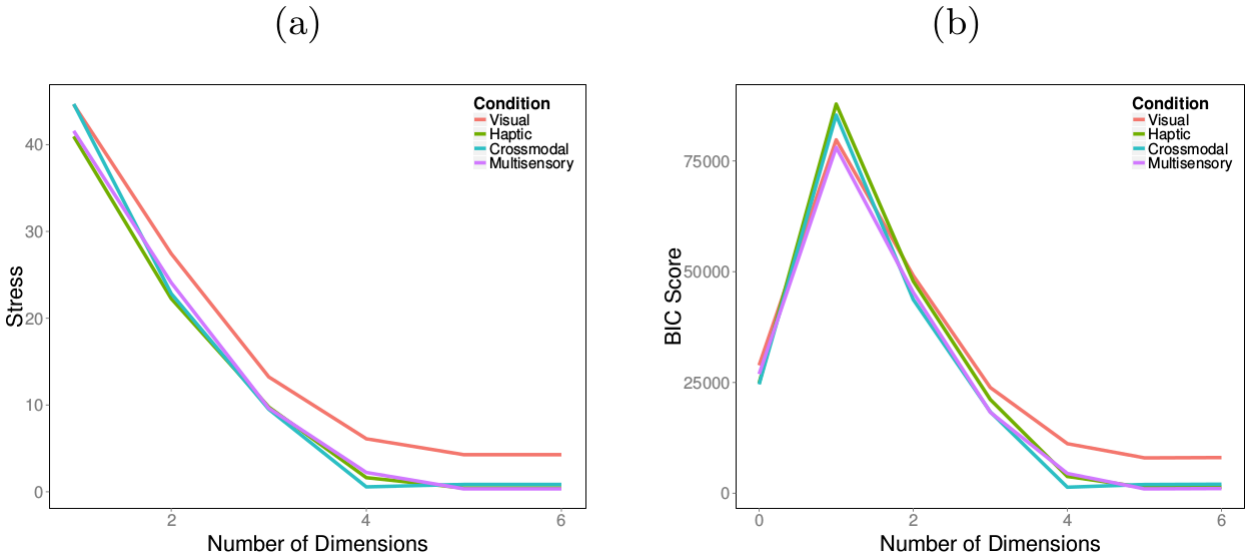
Results from MDS analysis. MDS (a) stress values and (b) BIC scores as a function of the number of dimensions.

**Fig 6:**
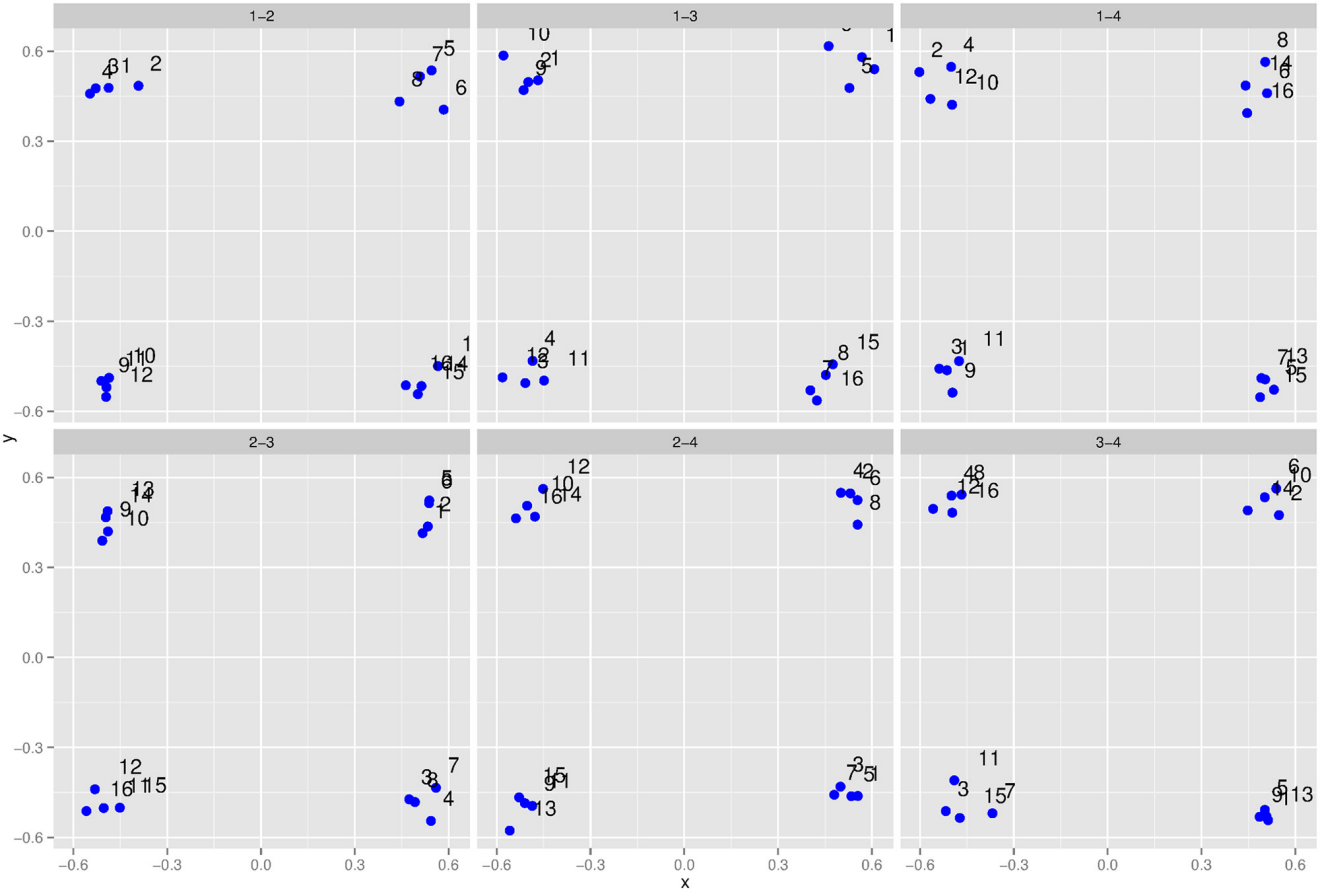
4D MDS space for the crossmodal condition. Each panel plots one of the six possible 2D projections of the 4D MDS space. Each point corresponds to one of the 16 objects.

Lastly, we analyzed subjects’ similarity ratings using non-metric multidimensional scaling (MDS) with the Manhattan distance function. Given a condition-level similarity matrix, MDS assigns locations in an abstract space to objects such that similar objects are nearby and dissimilar objects are far away [64–66]. To evaluate the dimensionality of this abstract space, we computed the “stress” value, a goodness-of-fit measure, for several different dimensionalities. In addition, we also calculated the Bayesian Information Criterion (BIC) score for each dimensionality. When using MDS, there are potential pitfalls when averaging similarity judgments of different subjects. If different subjects use different abstract spaces, then averaging will lose this information. In addition, average similarity ratings can be fit well by MDS regardless of the nature of individual subject’s ratings due to the increased symmetry of the average ratings [67]. Lee and Pope [68] developed a BIC score that ameliorates these potential pitfalls. This score takes into account both the fit and complexity of an MDS model. The results based on stress values and BIC scores are shown in Fig 5a and 5b, respectively. In both cases, values typically reach a minimum (or nearly so) at four dimensions in all experimental conditions. In Fig 6, we plot the MDS space with four dimensions for the crossmodal condition. The results for other conditions are omitted since they are all qualitatively quite similar. In each panel of Fig 6, we plot two of the four dimensions against each other, i.e., project the 4D space down to 2D. What is striking is the clear clustering in all panels. We see four clusters of four objects where each dimension takes one of two possible values. This is precisely the list-of-parts representation found by the Bayesian nonparametric additive clustering technique.

In summary, our correlational analyses of the experimental data reveal that subjects made similar similarity judgments in visual, haptic, crossmodal, and multisensory conditions. This indicates that subjects’ judgments were modality invariant. Our analyses using a Bayesian nonparametric additive clustering technique and using multidimensional scaling indicate that subjects formed the same set of modality-independent object representations in all conditions.

#### Simulation results

Here we evaluate whether the MVH model provides a good account of our experimental data. To conduct this evaluation, however, the model must be supplemented with an object similarity metric. Such a metric could potentially take several different forms. For example, object similarity could be computed based on modality-independent features. Alternatively, it could be based on modality-specific features such as visual or haptic features.

Researchers studying how people represent space have made a surprising discovery. Spatial locations can be represented in many different reference frames, such as eye-centered, head-centered, body-centered, or limb-position centered coordinate systems. Counterintuitively, people often transform representations of spatial locations into a common reference frame, namely an eye-centered reference frame, when planning and executing motor movements [69–72].

These studies raise an interesting issue: In what reference frame do people judge object similarity? Do they judge object similarity in a modality-independent feature space? Or do they judge object similarity in a sensory-specific feature space such as a visual or haptic space? Here we address these questions by augmenting the MVH model with different object similarity functions.

The hypothesis that people’s percepts are modality invariant predicts that people judge object similarity based on the values of modality-independent features. An alternative possibility is that people acquire modality-independent object representations when objects are viewed and/or grasped, but then re-represent objects in terms of visual features for the purpose of judging object similarity. The mapping from modality-independent to visual features could be achieved by a vision-specific forward model. A second alternative is that people re-represent objects in terms of haptic features (via a haptic-specific forward model) to judge object similarity. Because the MVH model includes modality-independent representations along with vision-specific and haptic-specific forward models, it can be used to evaluate these different possibilities.

In one set of simulations, the model was used to compute object similarity in a modality-independent feature space. On each simulated trial, the model computed modality-independent representations for two objects. Next, the objects’ similarity was estimated using a tree-based similarity measure known as “tree edit distance”[73]. In brief, this measure has a library of three tree-based operators: rename node, remove node, and insert node. Given two modality-independent object representations—that is, two spatial trees or MAP estimates of the shapes of two objects—this similarity measure counts the number of operators in the shortest sequence of operators that converts one representation to the other representation (or vice versa). For similar representations, the representation for object *A* can be converted to the representation for object *B* using a short operator sequence, and thus these representations have a small distance. For dissimilar representations, a longer operator sequence is required to convert one object representation to the other, and thus these representations have a large distance. In our simulations, we first placed the object representations in a canonical form, and then measured pairwise distances between objects using the tree edit distance measure of Zhang and Shasha [73].

In a second set of simulations, the model was used to compute object similarity in a visual feature space. As above, the model was used to acquire modality-independent representations for two objects on each simulated trial. Next, the vision-specific forward model was used to map each object representation to images of the represented object, thereby re-representing each object from a modality-independent reference frame to a visual reference frame. Given three images from orthogonal viewpoints of each object (see Fig 4a), the similarity of the two objects was estimated as the Euclidean distance between the images of the objects based on their pixel values.

In a final set of simulations, the model was used to compute object similarity in a haptic feature space. This set is identical to the set described in the previous paragraph except that the haptic-specific forward model (GraspIt!) was used to map each object representation to sets of a simulated hand’s joint angles, thereby re-representing each object from a modality-independent reference frame to a haptic frame. Given sets of joint angles for each object, the similarity of two objects was estimated as the Euclidean distance between the haptic features of the objects based on their associated joint angles.

**Fig 7:**
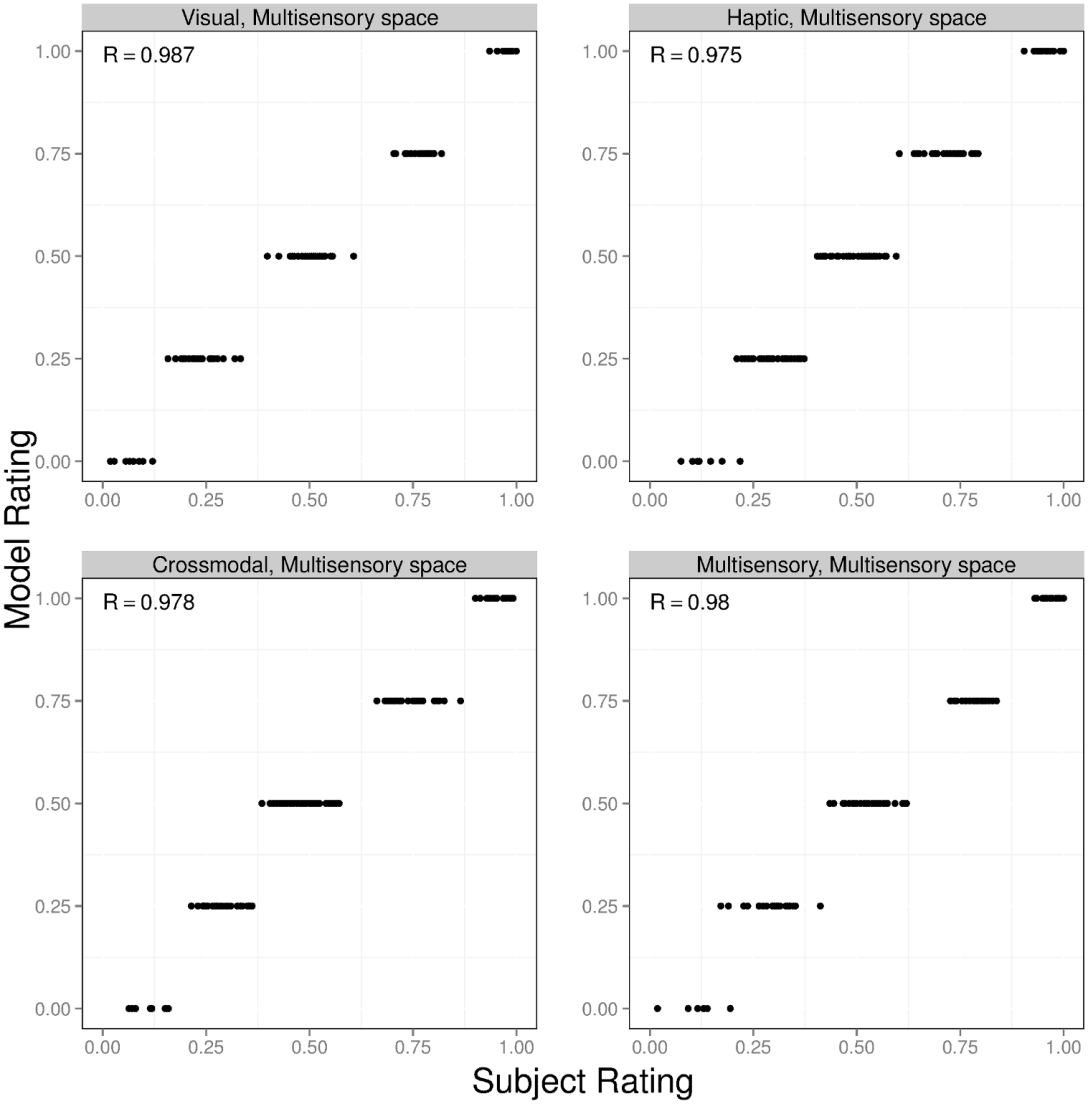
Results for the MVH-M model (this model computes object similarity in a modality-independent feature space). The four graphs correspond to the visual (top left), haptic (top right), crossmodal (bottom left), and multisensory (bottom right) experimental conditions. The horizontal axis of each graph shows subjects’ object similarity ratings (averaged across all subjects, and linearly scaled to range from 0 to 1). The vertical axis shows the model’s similarity ratings (linearly scaled to range from 0 to 1). The correlation (denoted *R*) between subject and model ratings is reported in the top-left corner of each graph. Note that MVH-M model’s similarity ratings take only a finite number of different values since parse trees are discrete structures, and therefore tree-edit distance returns only integer values.

**Fig 8:**
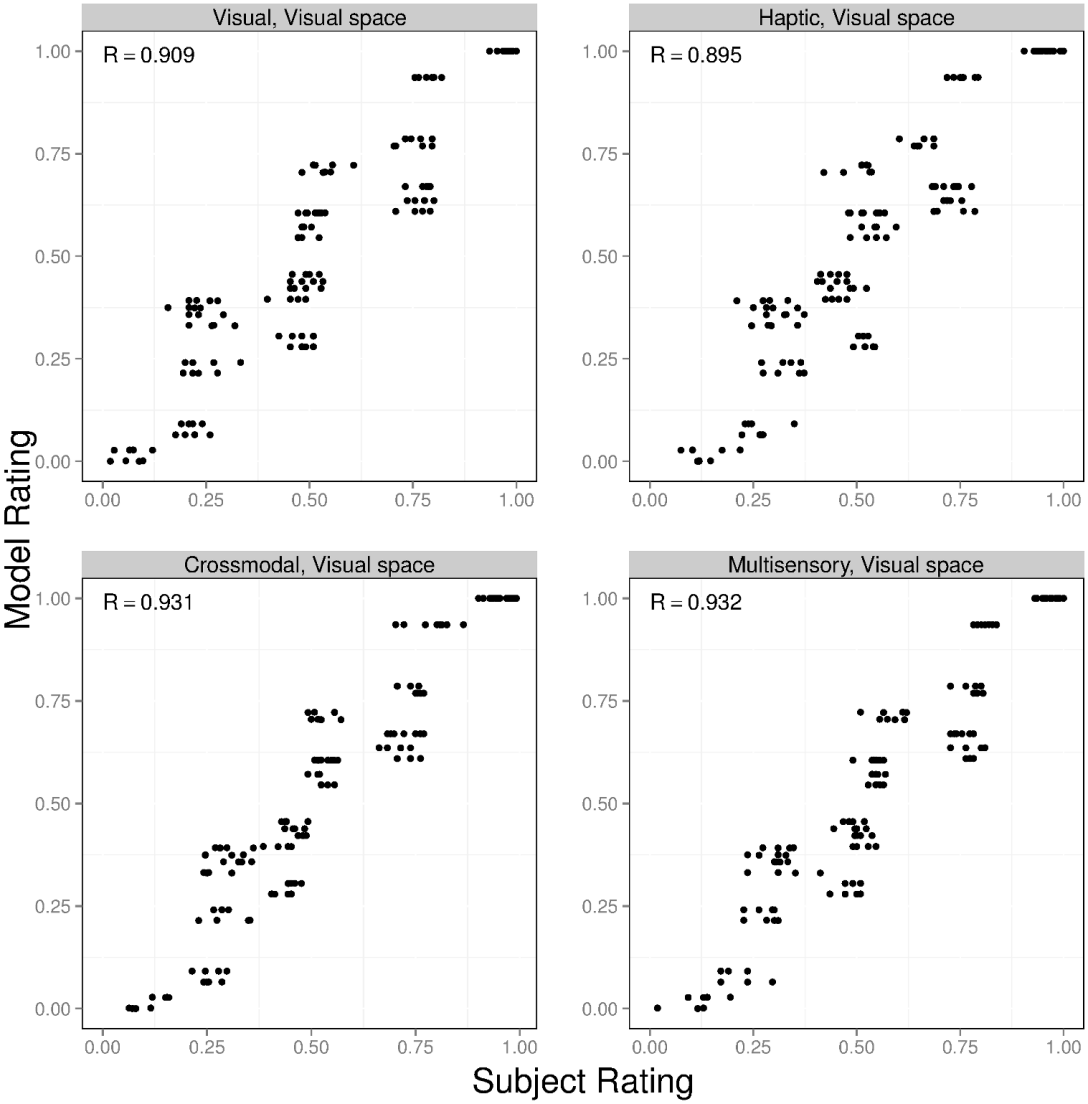
Results for the MVH-V model (this model computes object similarity in a visual feature space). The format of this figure is identical to the format of Fig 7.

**Fig 9:**
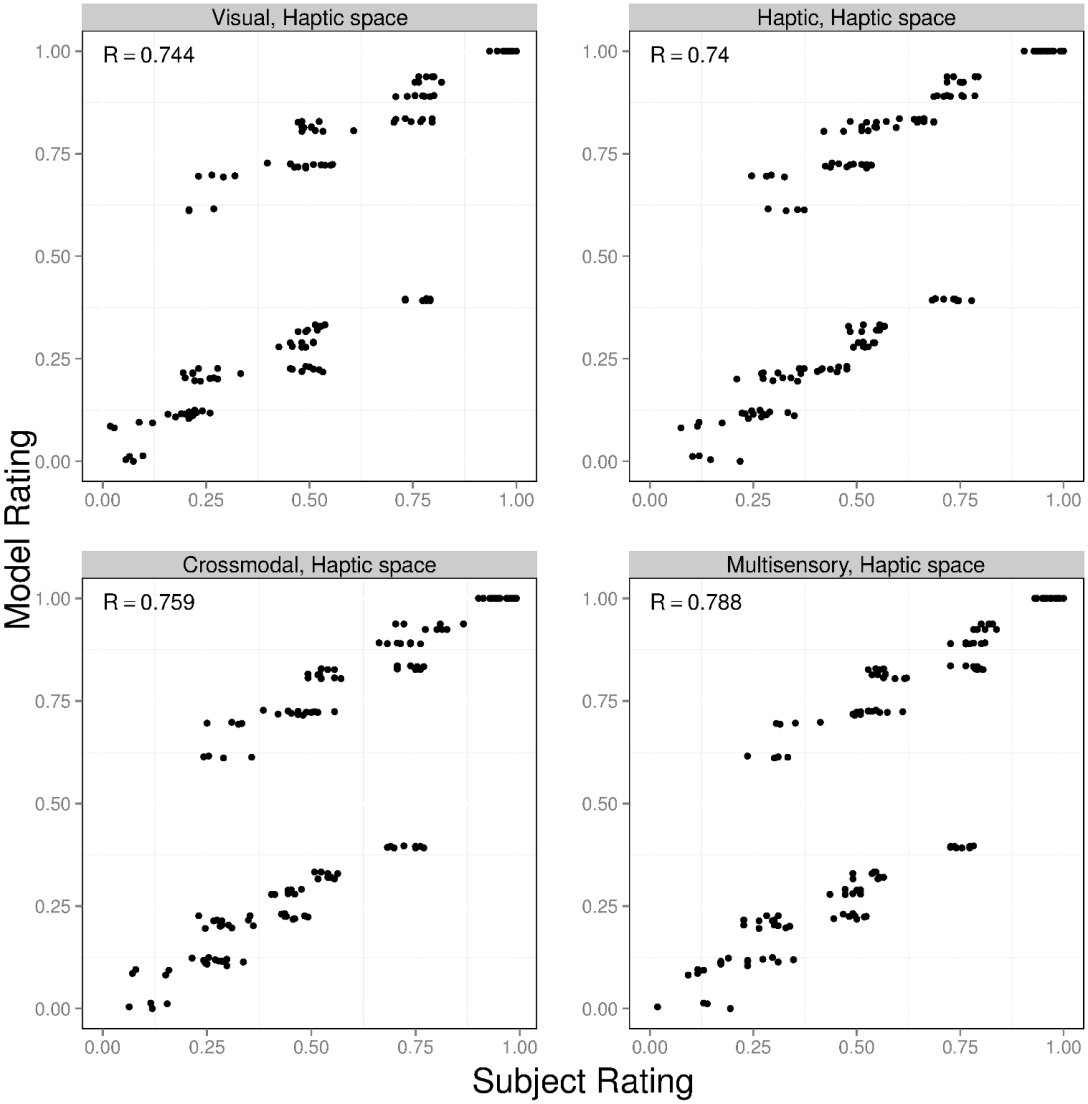
Results for the MVH-H model (this model computes object similarity in a haptic feature space). The format of this figure is identical to the format of Fig 7.

Which set of simulations produced object similarity ratings matching the ratings provided by our experimental subjects? For ease of explanation, we refer to the model augmented with the modality-independent based, visual-based, and haptic-based similarity functions as the MVH-M, MVH-V, and MVH-H models, respectively. The results for these three models are shown in Figs 7, 8 and 9. In each figure, the four graphs correspond to the visual, haptic, crossmodal, and multisensory conditions. The horizontal axis of each graph shows subjects’ object similarity ratings (averaged across all subjects, and linearly scaled to range from 0 to 1). The vertical axis shows a model’s similarity ratings (linearly scaled to range from 0 to 1). Each graph contains 136 points, one point for each possible pair of objects. The correlation (denoted *R*) between subject and model ratings is reported in the top-left corner of each graph.

A comparison of these figures reveals that the object similarity ratings of the MVH-M model provide an excellent quantitative fit to subjects’ ratings. Indeed, the correlation *R* ranges from 0.975 to 0.987 across the different experimental conditions (explaining 95%–97% of the variance in ratings). In other words, the MVH-M model provides a (nearly) perfect account of our experimental data. The MVH-V model provides a reasonably good fit to subjects’ data, though this fit is not as good as the fit provided by the MVH-M model. Based on a two-tailed *t*-test using the Fisher *z*-transformation, correlations for the MVH-M model are always greater than the corresponding correlations for the MVH-V model (p < 0.05). In addition, correlations for the MVH-M model and the MVH-V model are always greater than those of the MVH-H model. That is, the MVH-M model performs best, followed by the MVH-V model, and then the MVH-H model.

In summary, we have compared the performances of three models. All models represent objects in a modality-independent manner. However, the models differ in the space in which they calculate object similarity. One model calculates similarity using modality-independent features (MVH-M), another model maps modality-independent features to visual features and calculates similarity on the basis of these visual features (MVH-V), and a final model maps modality-independent features to haptic features and calculates similarity on the basis of these haptic features (MVH-H). Our results show that the MVH-M model’s similarity ratings provide the best quantitative fit to subjects’ ratings. Consequently, we hypothesize that subjects computed object similarity in a modality-independent feature space. That is, subjects acquired modality-independent object shape representations based on visual signals, haptic signals, or both, and then compared two objects’ shape representations in order to judge their similarity.

## Discussion

This paper has studied the problem of learning modality-independent, conceptual representations from modality-specific sensory signals. We hypothesized that any system that can accomplish this feat will include three components: a representational language for characterizing modality-independent representations, a set of sensory-specific forward models for mapping from modality-independent representations to sensory signals, and an inference algorithm for inverting forward models (i.e., an algorithm for using sensory signals to infer modality-independent representations).

To evaluate our theoretical framework, we instantiated it in the form of a computational model that learns object shape representations from visual and/or haptic signals. The model uses a probabilistic context-free grammar to characterize modality-independent representations of object shape, uses a computer graphics toolkit (VTK) and a human hand simulator (GraspIt!) to map from object representations to visual and haptic features, respectively, and uses a Bayesian inference algorithm to infer modality-independent object representations from visual and/or haptic signals. Simulation results show that the model infers identical object representations when an object is viewed, grasped, or both. That is, the model’s percepts are modality invariant. It is worth pointing out that the particular implementational choices we have made in our model are in some sense arbitrary; any model that instantiates our framework will be able to capture modality invariance. Therefore, from this perspective, our particular model in this work should be taken as one concrete example of how modality independent representations can be acquired and used.

Our work in this paper focused on showing how our framework can capture one aspect of multisensory perception, i.e., modality invariance. We take this as an encouraging first step in applying our framework to multisensory perception more generally. We believe other aspects of multisensory perception (such as cue combination, crossmodal transfer of knowledge, and crossmodal recognition) can be easily understood and treated in our framework.

The paper also reported the results of an experiment in which different subjects rated the similarity of pairs of objects in different sensory conditions, and showed that the model provides a very good account of subjects’ ratings. Our experimental results suggest that people extract modality independent shape representations from sensory input and base their judgments of similarity on such representations. The success of our model in accounting for these results are important from two perspectives. First, from a larger perspective, it is significant as a validation of our theoretical framework. Second, it constitutes an important contribution to cognitive modeling, particularly an emerging probabilistic language-of-thought approach, by showing how symbolic and statistical approaches can be combined in order to understand aspects of human perception.

### Related research

Our theoretical framework is closely related to the long standing vision-as-inference [74] approach to visual perception. In this approach, the computational problem of visual perception is formalized as the inversion of a generative process; this generative process specifies how the causes in the world, e.g., objects, give rise to 2D images on the retina. Then, the purpose of the visual system is to invert this generative model to infer the most likely causes, i.e., the explanation, for the observed sensory data. This approach, also called analysis-by-synthesis, has featured prominently both in cognitive science [75, 76] and computer vision [77–79]. Our work here can be seen as the application of this approach to multisensory perception.

Previous research has instantiated our general theoretical framework in other ways. For example, Yildirim and Jacobs [80] developed a latent variable model of multisensory perception. In this model, modality-independent representations are distributed representations over binary latent variables. Sensory-specific forward models map the modality-independent representations to sensory (e.g., visual, auditory, haptic) features. The acquisition of modality-independent representations takes place when a Bayesian inference algorithm (the Indian Buffet Process [63]) uses the sensory features to infer these representations. Advantages of this model include the fact that the dimensionality of the modality-independent representations adapts based on the complexity of the training data set, the model learns its sensory-specific forward models, and the model shows modality invariance. Disadvantages include the fact that the inferred modality-independent representations (distributed representations over latent variables) are difficult to interpret, and the fact that the sensory-specific forward models are restricted to being linear. Perhaps its biggest disadvantage is that it requires a well-chosen set of sensory features in order to perform well on large-scale problems. In the absence of good sensory features, it scales poorly, mostly due to its linear sensory-specific forward models and complex inference algorithm.

As a second example, Yildirim and Jacobs [2] described a model of visual-haptic object shape perception that is a direct precursor to the MVH model described in this paper. Perhaps its biggest difference with the model presented here is that it represents parts as generalized cylinders, and parts connect to each other using a large number of “docking locations”. This strategy for representing object shape provides enormous flexibility, but this flexibility comes at a price. Inference using this model is severely underconstrained. Consequently, the investigators designed a customized (i.e., *ad hoc*) Bayesian inference algorithm. Despite the use of this algorithm, inference is computationally expensive. That is, like the latent variable model described in the previous paragraph, the model of Yildirim and Jacobs [2] scales poorly.

### Probabilistic language-of-thought

We believe that the MVH model described in this paper has significant theoretical and practical advantages over alternatives. These arise primarily due to its use of a highly structured implementation of a representational language for characterizing modality-independent representations. In particular, the model combines symbolic and statistical approaches to specify a probabilistic context-free object shape grammar. Due to this shape grammar, the model is able to use a principled inference algorithm that has previously been applied to probabilistic grammars in other domains. We find that inference in the model is often computationally tractable. We are reasonably optimistic that the model (or, rather, appropriately extended versions of the model) will scale well to larger-scale problems. Although important challenges obviously remain, our optimism stems from the fact that shape grammars (much more complex than the one reported here) are regularly used in the Computer Vision and Computer Graphics literatures to address large-scale problems. In addition, due to its principled approach, the model should be easy to extend in the future because relationships between the model and other models in the Cognitive Science and Artificial Intelligence literatures using grammars, such as models of language, are transparent. As a consequence, lessons learned from other models will be easy to borrow for the purpose of developing improved versions of the model described here.

In Cognitive Science, there are many frameworks for cognitive modeling. For example, one school of thought favors symbolic approaches, such as approaches based on grammars, production rules, or logic. An advantage of symbolic approaches is their rich representational expressiveness—they can often characterize a wide variety of entities in a compact and efficient manner. A disadvantage of these approaches is that they are often “brittle” when used in noisy or uncertain environments. An alternative school of thought favors statistical approaches, such as approaches based on neural networks or Bayesian inference. An advantage of statistical approaches is their ability to learn and adapt, and their robustness to noise and uncertainty. Their main disadvantage is that they often require highly structured prior distributions or likelihood functions to work well [81]. Advocates of symbolic and statistical schools of thought have often engaged in heated debates [82–85]. Unfortunately, these debates have not led to a resolution as to which approach is best.

A recently emerging viewpoint in the Cognitive Science literature is that both symbolic and statistical approaches have important merits, and thus it may be best to pursue a hybrid framework taking advantage of each approach’s best aspects [45–48]. This viewpoint is referred to here as a “probabilistic language of thought” approach because it applies probabilistic inference to a representation consisting of symbolic primitives and combinatorial rules [86]. To date, the probabilistic language-of-thought approach has been used almost exclusively in domains that are typically modeled using symbolic methods, such as human language and high-level cognition. A significant contribution of the research presented here is that it develops and applies this approach in the domain of perception, an area whose study is dominated by statistical techniques.

### Future research

We foresee at least three areas of future research. First, the framework described here sheds light on modality invariance. Future work will need to study whether this framework also sheds light on other aspects of multisensory perception and cognition. For example, can the framework be used to understand why our percepts based on two modalities are often more accurate than our percepts based on a single modality, why training with two modalities is often superior to training with a single modality (even when testing is conducted in unisensory conditions), or why crossmodal transfer of knowledge is often, but not always, successful? Future work will also need to study the applicability of the framework to other sensory domains, such as visual and auditory or auditory and haptic environments. Future work will also need to consider how our framework can be extended to study the acquisition of other types of conceptual knowledge from sensory signals.

Second, future research will need to study the role of forward models in perception and cognition. For example, we have speculated that sensory-specific forward models may be ways of implementing sensory imagery, and thus our framework predicts a role for imagery in multisensory perception. Behavioral, neurophysiological, and computational studies are needed to better understand and evaluate this hypothesis. From a technological perspective, it is advantageous that we live in a “golden age” of forward models. New and improved forward models are frequently being reported in the scientific literature and made available on the world wide web (e.g., physics engines providing approximate simulations of physical systems such as rigid body dynamics or fluid dynamics). These forward models will allow cognitive scientists to study human perception, cognition, and action in much more realistic ways than has previously been possible.

Finally, cognitive scientists often make a distinction between rational models and process models [87]. Rational models (or computational theories [88]) are models of optimal or normative behavior, characterizing the problems that need to be solved in order to generate the behavior as well as their optimal solutions. In contrast, process models (or models at the “representation and algorithm” level of analysis [88]) are models of people’s behaviors, characterizing the mental representations and operations that people use when generating their behavior. Because the MVH model’s inference algorithm is optimal according to Bayesian criteria, and because this algorithm is not psychologically plausible, the model should be regarded as a rational model, not as a process model. Nonetheless, we believe that there are benefits to regarding the MVH model as a rational/process hybrid. Like rational models, the MVH model is based on optimality considerations. However, like process models, it uses psychologically plausible representations and operations (e.g., grammars, forward models).

For readers solely interested in process models, we claim that the MVH model is a good starting point. As pointed out by others [89, 90], the MCMC inference algorithm used by the MVH model can be replaced by approximate inference algorithms (known as particle filter or sequential Monte Carlo algorithms) that are psychologically plausible. Doing so would lead to a so-called “rational process model”, a type of model that is psychologically plausible and also possesses many of the advantages of rational models. Future work will need to study the benefits of extending our framework through the use of psychologially plausible and approximately optimal inference algorithms to create rational process models of human perception.

## Methods

### Ethics statement

The experiments were approved by the Research Subjects Review Board of the University of Rochester. All subjects gave informed consent.

### Multisensory-Visual-Haptic (MVH) model

#### Shape grammar

**Fig 10:**
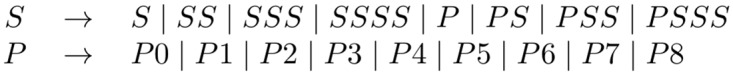
Production rules of the shape grammar in Backus-Naur form. S denotes spatial nodes, and P refer to part nodes. S is also the start symbol of the grammar. P1, P2, etc. are the object parts as seen in Fig 1.

The production rules of the MVH model’s shape grammar are shown in Fig 10. The grammar is an instance of a probabilistic context-free grammar. However, probabilities for each production rule are not shown in Fig 10 because our statistical inference procedure marginalizes over the space of all probability assignments (see below). Production rules characterize the number of parts and the specific parts comprising an object. The rules contain two non-terminal symbols, S and P. Non-terminal P is always replaced by a terminal representing a specific object part. Non-terminal S is used for representing the number of parts in an object. Production rules are supplemented with additional information characterizing the spatial relations among parts.

An object is generated using a particular sequence of production rules from the grammar. This sequence is known as a derivation which can be illustrated using a parse tree. To represent the spatial relations among object parts, a parse tree is extended to a spatial tree. Before describing this extension, it will be useful to think about how 3-D space can be given a multi-resolution representation. At the coarsest resolution in this representation, a “voxel” corresponds to the entire space. The center location of this voxel is the origin of the space, denoted (0, 0, 0). At a finer resolution, this voxel is divided into 27 equal sized subvoxels arranged to form a 3 × 3 × 3 grid. Using a Cartesian coordinate system with axes labeled x, y, and z, a coordinate of a subvoxel’s location along an axis is either −1, 0, or 1. For example, traversing the z-axis would reveal subvoxels located at (−1, −1, −1), (−1, −1, 0), and (−1, −1, 1). This process can be repeated. For instance, the subvoxel at (−1, −1, −1) can be divided into 27 subsubvoxels. The coordinates of subsubvoxels would also be either −1, 0, or 1. Note that the location of a subsubvoxel is relative to the location of its parent subvoxel which, in turn, is relative to the location of its parent voxel.

**Fig 11:**
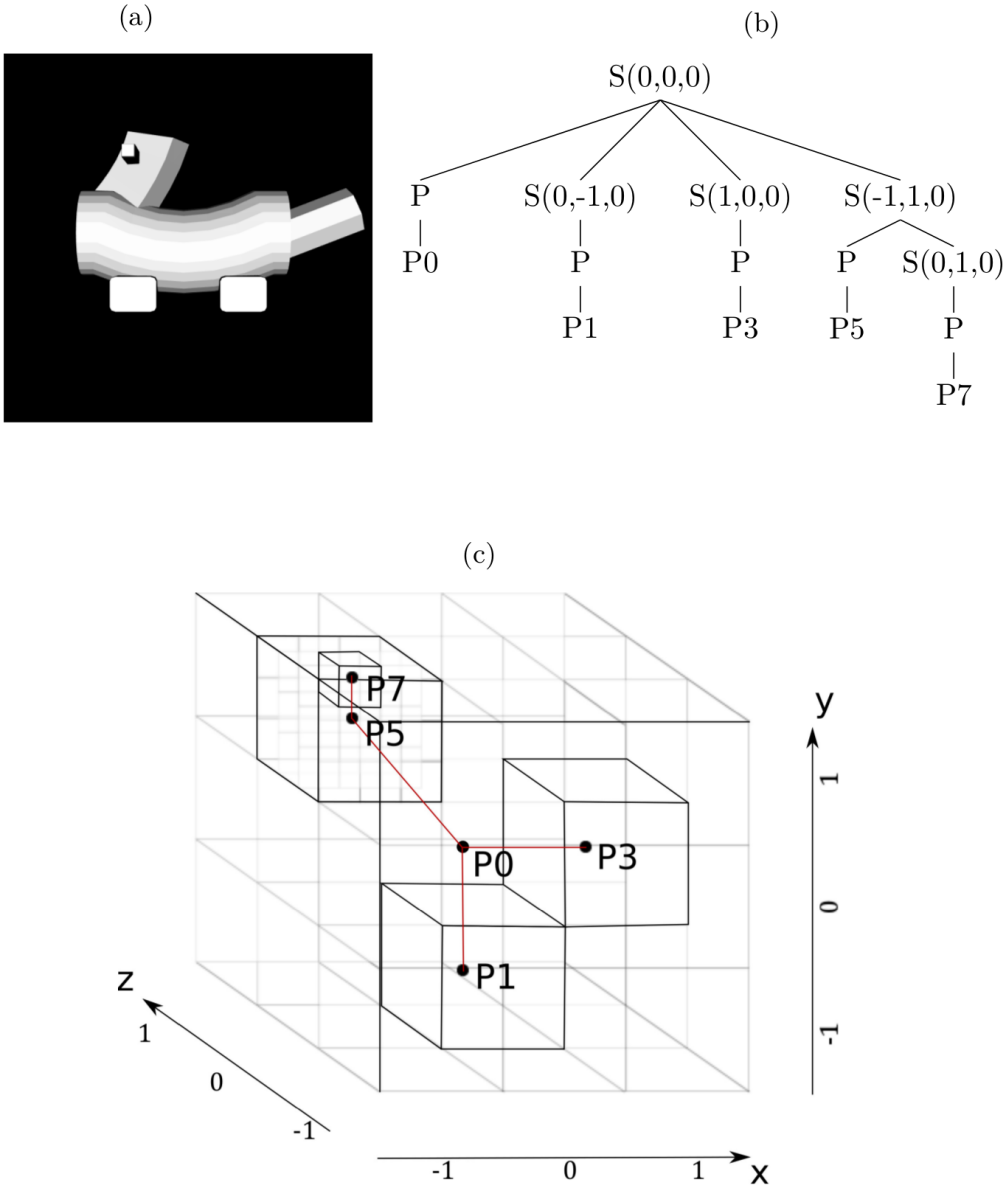
Illustration of the multi-resolution representation of 3-D space. (a) Image of an object. (b) Spatial tree representing the parts and spatial relations among parts for the object in (a). (c) Illustration of how the spatial tree uses a multi-resolution representation to represent the locations of object parts.

The addition of multi-resolution spatial information to a parse tree converts this tree to a spatial tree. This process is illustrated in Fig 11. Consider the object shown in Fig 11a and the spatial tree for the derivation of this object shown in Fig 11b. The root S node is associated with a voxel centered at the origin (0, 0, 0) of the 3-D space. This node is expanded using the rule *S* → *PSSS*, and locations are assigned to the subvoxels associated with the S nodes [in the figure, these locations are (0, −1, 0), (1, 0, 0), and (−1, 1, 0), respectively]. The P node is replaced with terminal P0 representing the cylindrical body (see Fig 1). This part is placed at the location of its grandparent S node. The two leftmost S nodes in the second level of the tree are eventually replaced with terminals P1 and P3, respectively. These parts are placed at the locations of their grandparent S nodes. The rightmost S node at the second level is expanded using the production *S* → *PS*, and a location is assigned to the S node [(0, 1, 0)]. The P node is replaced with terminal P5. The final S node is eventually replaced with terminal P7.

The multi-resolution representation of 3-D space, and the placement of parts in this space is illustrated in Fig 11c. Two facts about spatial trees are evident from this figure. First, smaller-sized voxels are reached as one moves deeper in a tree, enabling the model to make finer-grained assignments of locations to object parts. Second, with the exception of the root S node, an S node is never associated with a voxel located at (0, 0, 0) because this would create a situation in which two parts are assigned the same location.

There are several properties of the model’s shape grammar and spatial trees that were chosen for convenience: (i) The maximum branching factor of the shape grammar is four; (ii) The creation of spatial trees through the addition of spatial information to parse trees is not strictly necessary. An equivalent representation could be achieved by a more complicated grammar with productions for all possible voxel coordinate assignments to child S nodes; and (iii) Without loss of generality, the set of possible object parts was chosen for convenience. In other situations, other sets could be selected (indeed, one could imagine a system that uses a segmentation algorithm to learn good sets). In addition, the fact that object parts are at fixed scales and orientations is not strictly necessary. More complicated spatial trees could allow for scaling and rotation of parts. Our point here is that the probabilistic shape grammar approach is general and powerful, though the full generality and power of this approach is not needed for our current purposes. Readers interested in how shape grammars can be used to characterize objects and scenes in more realistic settings should consult the Computer Vision and Computer Graphics literatures [35–42].

#### Prior distribution over object representations

An object representation consists of two components, a parse tree, denoted T, and a spatial model, denoted S. The prior probability for an object representation is defined as:

$$\begin{array}{c} P(T,S|G)=P(T|G)P(S|T) \end{array}$$ (1)

where G denotes the shape grammar.

Due to the nature of our grammar, an object has a unique derivation, and thus a unique parse tree. Recall that a derivation is a sequence of productions in the shape grammar that ends when all non-terminals are replaced with terminals. At each step of a derivation, a choice is made among the productions which could be used to expand a non-terminal. Because a probability is assigned to each production choice in a derivation, the probability of the complete derivation is the product of the probabilities for these choices. That is, the probability of a parse tree is:

$$\begin{array}{c} P\left(T|G,\rho \right)=\prod_{n\in N_{nt}}P\left(n\to ch\left(n\right)|G,\rho \right) \end{array}$$ (2)

where $N_{nt}$ is the set of non-terminal nodes in the tree, *ch*(*n*) is the set of node *n*’s children nodes, and P(n→ch(n)|G,ρ) is the probability for production rule *n* → *ch*(*n*). In this equation, *ρ* denotes the set of probability assignments to production rules. Allowing for uncertainty in these production probabilities, we integrate over *ρ*:

$$\begin{array}{c} P(T|G)=\int P(T|G,\rho )P(\rho |G)d\rho . \end{array}$$ (3)

Because there is no reason to prefer any specific set of production probabilities, we assume that P(ρ|G) is a uniform distribution. With this assumption, the integral has a Multinomial-Dirichlet form, and thus can be solved analytically:

$$\begin{array}{c} P\left(T|G\right)=\prod_{s\in G_{nt}}\frac{\beta (C(T,s)+1)}{\beta (1)}. \end{array}$$ (4)

Here, $G_{nt}$ is the set of non-terminal symbols in grammar G, *β*(⋅) is the multinomial beta function, **1** is a vector of ones, and C(T,s) is a vector of counts of the productions for non-terminal *s* in parse tree T (the count of a rule increments each time the rule is used).

An advantage of this distribution over parse trees is that it favors “simple” trees, meaning trees corresponding to short derivations. (To see this, note that Eq 2 multiplies probabilities [numbers less than one]. The number of terms that are multiplied increases with the length of the derivation.) Consequently, it can be regarded as a type of Occam’s Razor.

In addition to the probability of parse tree T, the calculation of the prior probability of an object representation also requires the probability of spatial model S (Eq 1). Recall that model S contains the voxel coordinates for each S node in a parse tree. Let V denote the set of possible voxel coordinates, a set with 26 elements (the 3 × 3 × 3 grid yields 27 subvoxels but the subvoxel centered at (0, 0, 0) is not a valid spatial assignment). Using $N_{S}$ to denote the set of S nodes in tree T, and assuming that all voxel coordinates are equally likely, the probability of model S is:

$$\begin{array}{c} P\left(S|T\right)=\prod_{n\in N_{S}}\frac{1}{\left|V\right|}=\frac{1}{{\left|V\right|}^{|N_{S}|}}. \end{array}$$ (5)

As above, this distribution favors spatial models associated with small parse trees, and thus is a type of Occam’s Razor.

#### Likelihood function

Recall that an object representation consists of a parse tree T and a spatial model S. Let *D* denote actual sensory data perceived by an observer, either visual features, haptic features, or both. Let F(T,S) denote predicted sensory features, predicted by the visual-specific forward model (VTK), the haptic-specific forward model (GraspIt!), or both. To define the likelihood function, we assume that perceived sensory data *D* is equal to predicted sensory features F(T,S) plus random noise distributed according to a Gaussian distribution:

$$\begin{array}{c} P\left(D|T,S\right)\propto \exp\left(-\frac{||D-{F\left(T,S\right)||}_{2}^{2}}{\sigma^{2}}\right) \end{array}$$ (6)

where *σ* ^2^ is a variance parameter.

#### MCMC algorithm

Using Bayes’ rule, the MVH model combines the prior distribution and the likelihood function to compute a posterior distribution over object representations:

$$\begin{array}{c} P(T,S|D,G)\propto P(D|T,S)P(S|T)P(T|G) \end{array}$$ (7)

where the three terms on the right-hand side are given by Eqs 6, 5 and 4, respectively. Unfortunately, exact computation of the posterior distribution is intractable. We, therefore, developed a Markov chain Monte Carlo (MCMC) algorithm that discovers good approximations to the posterior.

MCMC is a family of methods for sampling from a desired probability distribution by constructing a Markov chain that has the desired distribution as its stationary distribution. A common MCMC method is the Metropolis-Hastings (MH) algorithm [91, 92]. This algorithm produces a sequence of samples. At each iteration, the algorithm picks a candidate for the next sample value based on the current sample value. With some probability, the candidate is accepted meaning that the candidate value is used in the next iteration or rejected meaning this value is discarded and the current value is reused in the next iteration.

In the context of our simulations, a value is a multisensory object representation—that is, a parse tree T and a spatial model S. At each iteration, our algorithm proposes a new representation, denoted $\left(T^{\prime },S^{\prime }\right)$, based on the current representation (T,S) with probability given by proposal distribution $q(T^{\prime },S^{\prime }|T,S)$. The new representation is accepted with a probability based on acceptance function $A(T^{\prime },S^{\prime };T,S)$.

We used two different proposal distributions in our simulations, one on even-numbered iterations and the other on odd-numbered iterations [93, 94]. The subtree-regeneration proposal distribution was originally developed by Goodman et al.[45]. When using this proposal distribution, a non-terminal node is randomly selected from parse tree T, all its descendants are removed, and new descendants are generated according to the rules of the shape grammar. Nodes removed from the parse tree are also removed from the spatial model, and random voxel coordinates are sampled for newly added nodes. The new representation is accepted with probability equal to the minimum of 1 and the value of an acceptance function:

$$\begin{array}{c} A\left(T^{\prime },S^{\prime };T,S\right)=\frac{P(D|T^{\prime },S^{\prime })}{P(D|T,S)}\frac{P(T^{\prime }|G)}{P(T|G)}\frac{|N_{nt}|}{|N_{nt}^{\prime }|}\frac{P(T|G,\rho )}{\left|P(T^{\prime }|G,\rho \right)} \end{array}$$ (8)

where $N_{nt}$ and $N_{nt}^{\prime }$ are the sets of all non-terminals in tree T and $T^{\prime }$, respectively.

**Fig 12:**
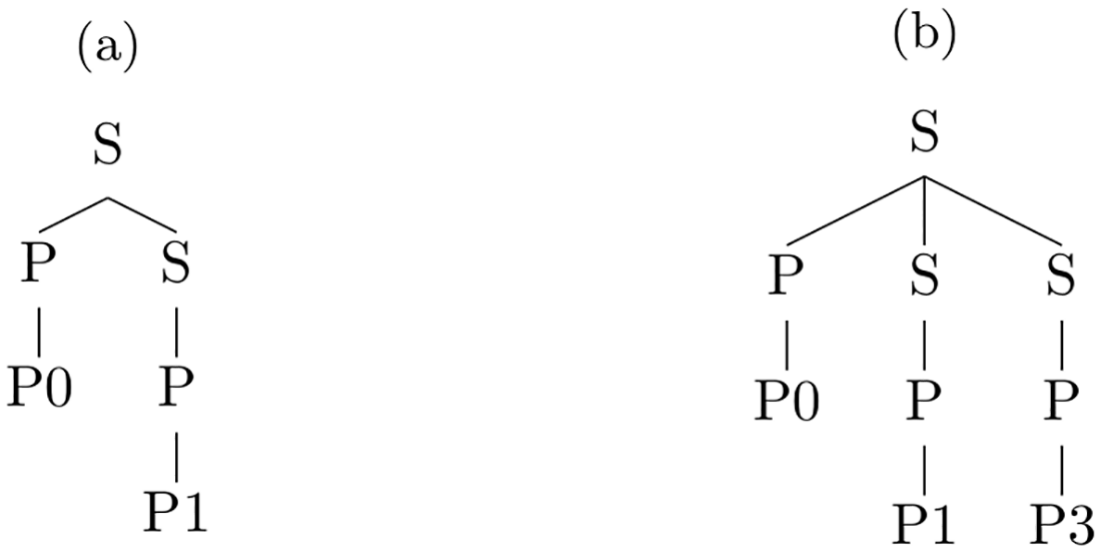
Parse trees for illustrating a difficulty with using the subtree-regeneration proposal. (a) Partially correct tree for a hypothetical example. (b) The “true” tree for the example. Note that it is impossible to propose the tree in (b) from the tree in (a) with a subtree-regeneration proposal without deleting and regenerating all the nodes.

Sole use of the subtree-regeneration proposal did not produce an efficient MCMC algorithm for our problem. This is mainly due to the fact that the algorithm sometimes proposes a new object representation which is very different from the current representation, thereby losing the desirable aspects of the current representation. Consider a scenario in which the current representation is partially correct, such as the parse tree in Fig 12a. Based on this tree, it is difficult to propose the more correct tree in Fig 12b without losing the desirable aspects of the current tree. To do so, the algorithm would have to choose the root node, thereby deleting nearly all of the current tree, and then generate the proposal tree nearly from scratch.

This observation led us to design the add/remove-part proposal. This proposal adds or removes object parts to a representation making it possible, for example, to propose the tree in Fig 12b based on the tree in Fig 12a, or vice versa. The proposal starts by randomly choosing whether to add or remove an object part. If adding a part, it draws a random part by choosing a terminal symbol from the grammar. Then it chooses an S node that has less than four children and adds a new S node as a child to the chosen S node. Finally, it expands the child S node to a P node and the P node to the chosen part. If removing a part, an S node that has a P node as its only child is chosen. This node and its descendants are removed. However, the proposal never chooses the root S node or an S node that is the only child of its parent as these will result in ungrammatical trees. The spatial model is updated accordingly. If a part is added, a random voxel coordinate is sampled for the newly added S node. If a part is removed, the corresponding S node (and its voxel coordinate) is removed. Assuming that representation $\left(T^{\prime },S^{\prime }\right)$ is proposed by adding a new part to (T,S), the new representation is accepted with probability equal to the minimum of 1 and the value of the acceptance function:

$$\begin{array}{c} A\left(T^{\prime },S^{\prime };T,S\right)=\frac{P(D|T^{\prime },S^{\prime })}{P(D|T,S)}\frac{P(T^{\prime }|G)}{P(T|G)}\frac{\left|A\right|}{|R^{\prime }|}\left|G_{t}\right| \end{array}$$ (9)

where $R^{\prime }$ is the set of *S* nodes in tree $T^{\prime }$ that can be removed, A is the set of *S* nodes in tree T to which a new child *S* node can be added, and $G_{t}$ is the set of terminal symbols in the grammar. Similarly, the acceptance function when removing a part is:

$$\begin{array}{c} A\left(T^{\prime },S^{\prime };T,S\right)=\frac{P(D|T^{\prime },S^{\prime })}{P(D|T,S)}\frac{P(T^{\prime }|G)}{P(T|G)}\frac{\left|R\right|}{|A^{\prime }\left|\right|G_{t}|} \end{array}$$ (10)

where R is the set of *S* nodes in tree T that can be removed, $A^{\prime }$ is the set of *S* nodes in tree $T^{\prime }$ to which a new child *S* node can be added.

It is easy to show that our algorithm is a valid Metropolis-Hastings sampler, meaning that it has the posterior distribution over multisensory object representations as its stationary distribution. Derivations for the acceptance functions for the subtree-regeneration and add/remove-part proposals are straightforward. Readers interested in these topics should contact the first author.

In our simulations, each MCMC chain was run for 10,000 iterations. Samples from the first 6,000 iterations were discarded as “burn-in”.

### Experimental Details

#### Stimuli

The experiment used the 16 objects in Fig 2. Visual stimuli consisted of images of objects rendered from a canonical (three-quarter) viewpoint so that an object’s parts and spatial relations among parts are clearly visible (Fig 2). Stimuli were presented on a 19-inch CRT computer monitor. Subjects sat approximately 55 cm from the monitor. When displayed on the monitor, visual stimuli spanned about 20 degrees in the horizontal dimension and 15 degrees in the vertical dimension. Visual displays were controlled using the PsychoPy software package [95].

Subjects received haptic inputs when they touched physical copies of the objects fabricated using a 3-D printing process (Fig 2). Physical objects were approximately 11.5 cm long, 6.0 cm wide, and 7.5 cm high. Subjects were instructed to freely and bimanually explore physical objects.

#### Procedure

On each experimental trial, a subject observed two objects and judged their similarity on a scale of 1 (low similarity) to 7 (high similarity). Within a block of 136 trials, each object was paired both with itself (16 trials) and with the other objects (each object could be paired with 15 other objects; ignoring order of object presentation [which was randomized], this results in 120 trials). Pairs were presented in random order. Subjects performed 4 blocks of trials.

The experiment included four conditions referred to as the visual, haptic, crossmodal, and multisensory conditions. Different groups of subjects were assigned to different conditions. We regard the crossmodal condition as the key experimental condition because it is the condition that directly evaluates the modality invariance of subjects’ percepts. The visual, haptic, and multisensory conditions are control conditions in the sense that data from these conditions are of interest primarily because they allow us to better understand results from the crossmodal condition.

In the visual condition, subjects saw an image of one object followed by an image of a second object. Images were displayed for 3.5 seconds.

In the haptic condition, physical objects were placed in a compartment under the computer monitor. The end of the compartment closest to a subject was covered with a black curtain. A subject could reach under the curtain to haptically explore an object. However, a subject could not view an object. Messages on the computer monitor and auditory signals indicated to a subject when she or he could pick up and drop objects. On each trial, an experimenter first placed one object in the compartment. The subject then haptically explored this object. The experimenter removed the first object and placed a second object in the compartment. The subject explored this second object. Each object was available for haptic exploration for 7 seconds. As is common in the scientific literature on visual-haptic perception, the haptic input in the haptic experimental condition was available for longer than the visual input in the visual condition [11, 60, 96, 97].

In the crossmodal condition, objects in a pair were presented in different sensory modalities. For one subgroup of three subjects, the first object was presented visually and the second object was presented haptically. For another subgroup of four subjects, this order was reversed. We checked for a difference in ratings between the two subgroups. A two-tailed Welch’s *t*-test (used when two samples have possibly unequal variances) did not find a significant effect of the order of the modalities in which objects were presented (t = 0.087, p = 0.935). We, therefore, grouped the data from these subgroups.

In the multisensory condition, both objects were presented both visually and haptically. During the 7 seconds in which an object could be touched, the visual image of the object was displayed for the final 3.5 seconds.

Visual and crossmodal conditions were run over two one-hour sessions on two different days, each session comprising two blocks of trials. For haptic and multisensory conditions, an individual block required about an hour to complete. These conditions were run over four one-hour sessions. Although subjects performed four blocks of trials, we discarded data from the first block because subjects were unfamiliar with the objects and with the experimental task during this block. Results reported above are based on data from blocks 2–4.

#### Subjects

Subjects were 30 students at the University of Rochester who reported normal or corrected-to-normal visual and haptic perception. Subjects were paid $10 per hour. Of the 30 subjects, 2 subjects provided similarity ratings that were highly inconsistent across blocks (one subject in the visual condition and the other in the multisensory condition). A Grubbs test [98] using each subject’s correlations among ratings in different blocks revealed that these two subjects’ ratings are statistical outliers (Subject 1: g = 2.185, p < 0.05; Subject 2: g = 2.256, p < 0.05). These ratings were discarded from further analyses. The remaining 28 subjects were divided among the four experimental conditions, seven subjects per condition.

#### MVH-V and MVH-H models applied to the experimental data

The MVH-V and MVH-H models are equivalent to alternative models. For instance, consider a model that computes object similarity based solely on the pixel values of images of those objects. In fact, this is equivalent to MVH-V. This equivalency arises from the fact the the MVH model’s MAP estimates of object shape are always correct (given an object, this estimate is the correct representation of the object in terms of the shape grammar). When MVH-V obtains images of two objects (by rendering the object representations using the vision-specific forward model), these images are also always correct (they are identical to the true images of the objects). Consequently, MVH-V performs no differently than a model that rates object similarity based on the pixel values of images of objects. Given this fact, why is MVH-V needed? It is because people do not always have images of two objects (consider a case where one object is viewed and the other object is grasped). Analogous remarks apply to MVH-H.
